# OHO: A Multi-Modal, Multi-Purpose Dataset for Human-Robot Object Hand-Over

**DOI:** 10.3390/s23187807

**Published:** 2023-09-11

**Authors:** Benedict Stephan, Mona Köhler, Steffen Müller, Yan Zhang, Horst-Michael Gross, Gunther Notni

**Affiliations:** 1 Neuroinformatics and Cognitive Robotics Lab, Technische Universität Ilmenau, 98693 Ilmenau, Germanyhorst-michael.gross@tu-ilmenau.de (H.-M.G.); 2 Group for Quality Assurance and Industrial Image Processing, Technische Universität Ilmenau, 98693 Ilmenau, Germanygunther.notni@tu-ilmenau.de (G.N.); 3 Fraunhofer Institute for Applied Optics and Precision Engineering, IOF Jena, 07745 Jena, Germany

**Keywords:** dataset, thermal image, semantic segmentation, hand-over, 6D pose estimation, automated labeling

## Abstract

In the context of collaborative robotics, handing over hand-held objects to a robot is a safety-critical task. Therefore, a robust distinction between human hands and presented objects in image data is essential to avoid contact with robotic grippers. To be able to develop machine learning methods for solving this problem, we created the OHO (Object Hand-Over) dataset of tools and other everyday objects being held by human hands. Our dataset consists of color, depth, and thermal images with the addition of pose and shape information about the objects in a real-world scenario. Although the focus of this paper is on instance segmentation, our dataset also enables training for different tasks such as 3D pose estimation or shape estimation of objects. For the instance segmentation task, we present a pipeline for automated label generation in point clouds, as well as image data. Through baseline experiments, we show that these labels are suitable for training an instance segmentation to distinguish hands from objects on a per-pixel basis. Moreover, we present qualitative results for applying our trained model in a real-world application.

## 1. Introduction

In the course of Industry 4.0, collaborative robots (cobots) are gaining more and more attention. For collaboration, successfully handing over objects between humans and cobots plays a major role. As possible injury to the human needs to be strictly avoided, the robot’s gripper should not touch the human hand. Therefore, the cobot needs to be aware of its surroundings and recognize objects of interest, as well as the human hand. To enable further processing steps, such as grasp planning and the execution of robotic motion trajectories, robust pixel-wise instance segmentation is required. State-of-the-art methods for instance segmentation such as Mask-RCNN [1] or PointRend [2] process RGB image data to achieve this goal. Recently, Transformer-based methods have outperformed CNN-based architectures. These models need a large amount of data or extensive pretraining to achieve comparable or better results [3]. If the necessary amount of training data are available, Transformers can be used as a replacement backbone for methods such as Mask-RCNN.

If additional depth data are available, the resulting pixel-wise segmentation mask can be utilized to reconstruct a labeled 3D scene for further usage, as described in [4]. Alternatively, a point cloud could be created from the RGB-D raw data, which can then be segmented using techniques such as PointNet [5], RandLA-Net [6], or SO-Net [7]. In [8], these and other point cloud segmentation methods were evaluated on the presented dataset.

In other applications, multi-spectral imaging has proven to be helpful when it comes to the segmentation of organic structures [9,10]. Therefore, we are interested in adapting the aforementioned methods designed for RGB images to incorporate multi-modal data, including thermal images. To incorporate depth information, architectures have been proposed that extract information from both depth and color images and fuse them at different stages within their backbone [11,12].

In addition to a method for generating instance segmentation masks, a suitable dataset is of major importance for both training and testing a model. There are several datasets with segmentation labels for hands [13,14,15,16,17,18,19], but few of these contain objects as well as labels. An overview of the datasets most similar to the dataset presented in this paper is presented in Table 1, and the sizes, modalities, and available label types in these datasets are also listed. HandNet [13] and the dataset presented in [14] contain depth and even thermal images but lack labeled objects. In contrast, the WorkingHands dataset [17] contains segmentation labels for objects but no thermal images. However, the available labels are merely semantic segmentation labels. Overlapping instances of the same category can, therefore, not be distinguished, which is a major requirement for grasping individual objects. Moreover, the majority of pixels in WorkingHands are labeled as void, which is an ill-defined class. This often leads to unnecessary object pixels predicted in the background (as seen in their results) and might be a problem for applications where it is necessary to locate individual objects.

Most similar to our dataset is the ContactPose [19] dataset. Even though the intention behind ContactPose is contact modeling between human hands and objects, it also includes instance segmentation labels for hands and objects. However, all objects are 3D-printed in blue, which limits its applicability to real-world instance segmentation. The only dataset that contains thermal images is the one of Kim et al. [14].

When collecting a dataset with the intended properties, one of the most time-consuming tasks is labeling. Ideally, either the recorded data or the setup allows for automated label generation. However, for good generalization, the collected data should still resemble real-world data. For example, the recording of thermal data in [14] allowed for the automated generation of instance segmentation labels for hands. However, the segmentation of the objects is just as important when objects are handed over to the cobot. Therefore, we additionally designed a recording setup so that the automated segmentation of hands and objects is easily possible while keeping the input data close to a real-world setting.

We want to emphasize that our automated labeling allows us to easily expand our dataset with new objects. This distinguishes our approach from those of other fixed datasets, which require manual labeling, as in WorkingHands [17]. In contrast to Kim et al. [14], we found that thermal images were not a useful basis for automated label generation. On the one hand, there are many warm background objects, and objects become warm in the hand during manipulation. On the other hand, thermal images have a much lower resolution than color images, which limits the label quality.

For good generalization of trained models, the diversity of training data is essential, which is realized through diverse augmentation techniques. We addressed this problem by recording the background and the hands with objects separately, which were then combined pairwise.

Due to the presented shortcomings of existing datasets in the context of our intended application, we collected a new multi-modal, multi-purpose dataset—named OHO—for human–robot Object Hand-Over. In this paper, we present the setup and collection process of the OHO dataset, as well as the automatic pipeline for generating instance segmentation labels. Then, we use the data to train state-of-the-art models in instance segmentation. Additionally, we implement the trained models in a real-world application to prove that the collected dataset is suitable for solving the instance segmentation task required for handing over objects. We created our dataset with other tasks in mind, such as 6D pose estimation and object shape reconstruction, which will be addressed in future work.

In summary, our main contributions are:

- A multi-modal dataset, including RGB, depth, and thermal data.
- A multi-purpose dataset for instance segmentation, point cloud segmentation, object shape, and pose estimation.
- A recording procedure and pipeline for automated instance segmentation labeling for hands and hand-held objects in 2D images, as well as 3D point clouds.
- Quantitative and qualitative baseline results for instance segmentation in 2D images.

## 2. Multi-Modal Object Hand-Over Dataset (OHO)

One goal of our ongoing research is the development of multi-modal sensors for safe human–robot interactions. Therefore, we want to compare different cameras and modalities, such as RGB, depth, and thermal data, in an application-relevant scenario.

**Figure 1:**
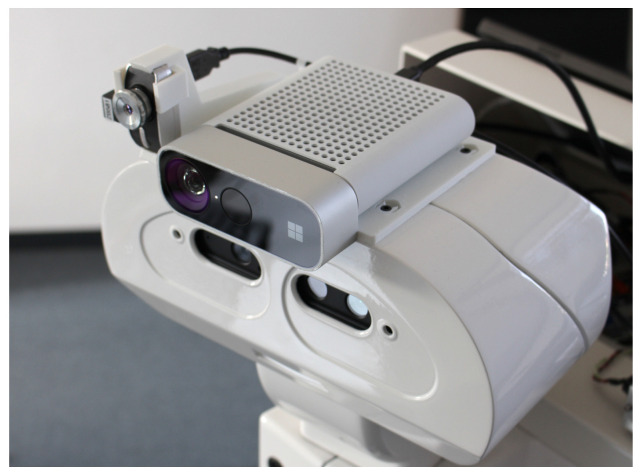
Cameras mounted on top of the TIAGo robot’s head.

**Figure 2:**
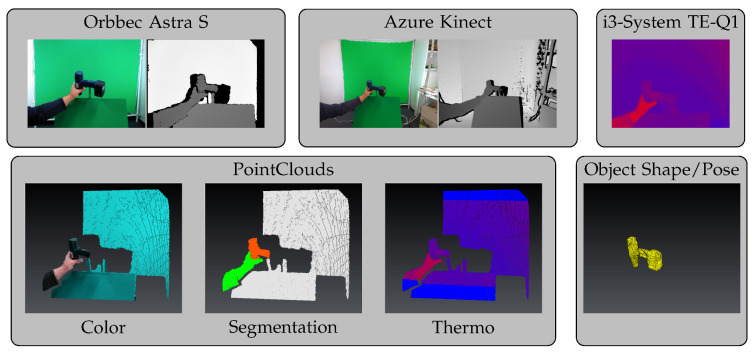
Examples of all modalities for one sample in our dataset, including color and depth images from two RGB-D cameras, a segmented point cloud (hand, object, and background), and object shape and pose, in addition to thermal data.

This means that we did not want to restrict our recordings to one type of RGB-D camera. Hence, we equipped our mobile robot, TIAGo, with an Azure Kinect and an i3-System TE-Q1 thermal camera (see Figure 1). Together with its internal Orbbec Astra S camera, we had a spectrum of cameras using different methods for depth perception (time of flight in Azure Kinect, and active stereo in Astra Orbbec). This resulted in three modalities in our dataset: color, depth, and thermal (and, indirectly, stereo images). Each sample in our dataset consisted of the following images, as shown in Figure 2: RGB images from two RGB-D cameras—Azure Kinect, with a resolution of 4096×3072, and Orbbec Astra S, with a resolution of 640×480—as well as the registered depth images from both cameras. Additionally, each sample contained a thermal image from an i3-System TE-Q1, with a resolution of 384×288.

For the selection of objects used in our dataset, we focused on handy items that a robot is able to grasp. Additionally, we captured shape data in the form of a triangle mesh for every recorded object by scanning them with a 3D scanner or modeling them in a CAD program. Moreover, for each sample, the relative poses of the cameras and the object (see Section 2.1), as well as the metadata for the scene, were generated. These included which hand (left or right) was holding the object and whether the object had been masked using masking tape to make it visible to the depth cameras. This was necessary for very dark or glossy metal objects (e.g., a black rubber hammer or the blade of a screwdriver), since both cameras had difficulties capturing depth data for some objects.

For recording, we used a green screen, which enabled automated label generation and foreground–background composition, as discussed in detail in Section 2.3. To replace the green screen background in every modality, we also recorded a set of backgrounds of 234 office scenes with the same camera configuration on the mobile robot. Afterward, the data were split into 171 backgrounds for training, 33 for validation, and 30 for testing.

### 2.1. Setup for Dataset Recording

The cameras had to be calibrated internally and externally to allow for the registration of thermal, depth, and RGB data. This was performed using a special checkerboard, which is visible in the binarized thermal image, as well as in the visual and near-IR images from the Azure Kinect and Astra Orbbec cameras. For details on the external calibration of the thermal and near-IR cameras, we refer the reader to [8], where along with the mathematics, the active calibration target is described precisely. Note that the Orbbec Astra S camera suffers from a thermal problem that affects the depth data over time. To compensate for this, a parametric transformation of the depth data was performed, which scaled the depth values by a factor that was linearly interpolated between two manually set parameters along the x-axis of the depth image. These scaling factors were manually set by visually aligning the resulting point clouds from both depth cameras.

**Figure 3:**
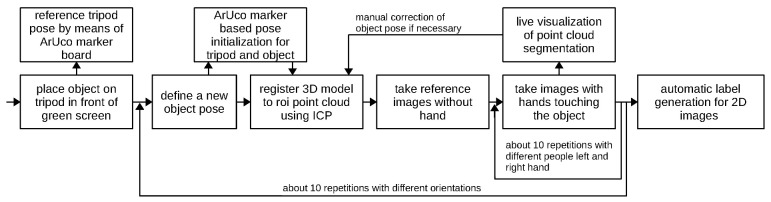
Sequence of recording one object for the OHO dataset.

As a prerequisite for potentially training pose estimation models and for our automatic labeling process, we needed the exact 6D pose of the object in the scene. Therefore, each object was fixed statically on a tripod in front of the green screen, and the point cloud was cropped so that only object points remained in the region of interest. The region of interest was defined relative to the pose of the tripod and was captured through the use of ArUco [20] markers on the tripod (see Figure 3 left). These markers were removed or covered by a green screen before recording the actual data. Note that the green screen was intentionally small to limit the reflections of green ambient light on the object. Afterward, we computed the object’s pose using an ICP (Iterative Closest Point) method [21], registering the given 3D model of the object to the point cloud of the Orbbec Astra S camera. The ArUco marker-based tracking of the tripod pose helped initialize the object pose after the pose had been changed. To minimize the required effort for recording, we used one object pose for multiple samples of a human hand holding the object. For each of the object poses, we recorded a reference image that only showed the object without a hand holding it. This has several advantages, such as providing useful information for automatic label generation (described later), having images of the single object for training a model that needs to be able to recognize the objects regardless of whether they are being held, and recomputing the object pose after the recording has taken place. After the reference image was taken, different people placed one of their hands on the fixed object and pretended to hold it. The hands in the images belong to seven different males and females. During recording, the point clouds were labeled automatically in real time, as described in Section 2.2. This allowed the operator to use the result as feedback to assess whether the setup and the current sample were correct. We made sure to manually check the results during recording and adjusted the ground-truth object poses when necessary.

Following this procedure, which is summarized in Figure 3, we recorded about 10 poses for each of the 43 objects in the 32 categories, where each object pose comprised about 10 different hand positions. This yielded a dataset of 5300 samples. We carefully split all the samples according to the object poses into training, validation, and test (one of the object poses for validation and one for test). Therefore, similar samples were not included in different splits.

### 2.2. Automated Labeling in Point Clouds

The first step in the automated labeling of the dataset was the annotation of point cloud points belonging to either the object or the human hand. Background points were segmented using a 3D region-of-interest box in relation to the fixed tripod position. Since we knew the 3D shape of the object and its pose, we computed the distances of all remaining point cloud points to the surface of the object model. By thresholding these distances (t=6 mm), a coarse segmentation of the object and hand points could be performed. Unfortunately, this resulted in some points on the hand that were close to the object being counted as object points. To compensate for this, the hand segments were dilated by 3–6 mm, depending on the current pose and object properties. This means that object points with a distance to a hand point smaller than the dilation radius were switched to hand points. In the end, we obtained a labeled point cloud, as shown in Figure 2, which can be used for either training point cloud segmentation methods or as a starting point for labeling color images, as described below. Note that the point cloud annotation only worked with the Orbbec Astra S point clouds, which used an active stereo approach. In contrast, the Azure Kinect point cloud did not produce usable segmentation results due to artifacts caused by abrupt changes in depth values. The time-of-flight depth image from the Azure Kinect contained interpolated points at object borders, which resulted in phantom 3D points in free space, making them difficult to filter out automatically. Thus, we used automated labeling of point clouds only for the images from the Orbbec Astra S data.

### 2.3. Automated Instance Segmentation Label Generation

To train a model to segment hands and objects, instance segmentation labels are required. Since it is time-consuming and, therefore, costly to label the samples by hand, an automated approach was used, leveraging the green screen and the recording of the reference samples of objects without hands. Our approach can be divided into two steps. First, a segmentation of the images into the foreground (hand and object) and the background is performed by removing the green screen. In the next step, the results of this foreground–background segmentation and the labeled point clouds (Section 2.2) projected onto the color image are used to compute the final instance segmentation labels with GrabCut [22]. These two steps are discussed in detail below.

To remove the green screen, we used the images from the Kinect Azure camera due to their better image quality, which made color keying easier. However, the generation pipeline described below can also be adjusted for use with color images recorded by the Orbbec Astra camera.

#### 2.3.1. Green Screen Removal

**Figure 4:**
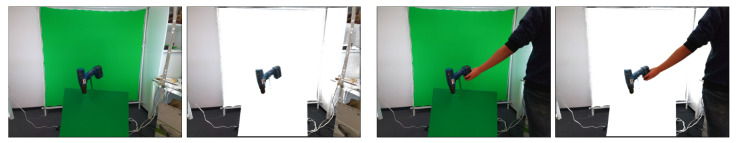
Example result of using the keying node in Blender on a reference recording (**left**) and the corresponding sample with a hand (**right**).

The first step in generating instance segmentation labels is to remove the green screen from the images to obtain a rough segmentation of the foreground—containing the hand and object—and the background. The free graphics software Blender (v2.82.7, https://www.blender.org/, accessed on 1 January 2020) comes with an implementation of green screen removal and the option to use it via a Python interface. For these reasons, the keying node in Blender was used to segment the foreground from the background. An example of the green screen removal results can be seen in Figure 4. Besides removing the green screen, this node is also capable of removing the green spill (green light reflected from the green screen and visible on the objects and hands). This is important for the images to be more realistic when replacing the background later, as described in Section 3. Because in our setup the green color of the fabric used in the background was slightly different than the green used to hide the mounting stand, we used two keying nodes in succession.

#### 2.3.2. GrabCut

Based on the green screen foreground segmentation and a projection of the segmented point cloud, we used GrabCut [22] to compute a detailed segmentation mask for the hand. In some cases, the skin color segmentation performed in Blender might be sufficient, but for most cases, this refinement is necessary to differentiate the fingers from the object because of shadows. With this hand mask, we subtracted the hand pixels from the foreground segmentation of the reference image to generate the final mask for the object in the sample image.

**Figure 5:**
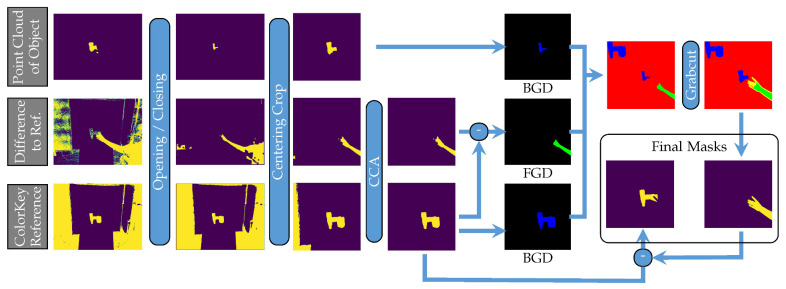
Pipeline for generation of segmentation labels. Color codes for GrabCut labels are as follows: BGD = blue, PR_BGD = red, PR_FGD = yellow, and FGD = green.

GrabCut performs the segmentation of an image into the foreground (in our case, the hand) and the background by formulating the segmentation as a graph cut problem, which aims to maximize the difference in the color histograms of foreground and background pixels. Therefore, we needed to first define prior label regions that specify the initial color histograms of the foreground and background. GrabCut differentiates four labels: foreground (FGD), probably foreground (PR_FGD), background (BGD), and probably background (PR_BGD). Figure 5 shows an overview of how the Blender results were used to assign parts of the image to these classes.

First, we preprocessed the foreground segmentations and the projected point cloud by applying opening and closing operations to remove segmentation artifacts. Then, the images were cropped around the center of the object to reduce computational complexity.

For removing static parts like the wall, which was not covered by the green screen, we computed a difference image between the reference recording and the recording containing the hand. After applying opening and closing operations on the difference image, we used a connected component analysis (CCA) to filter out the remaining small components, leaving only the hand and object areas. For the reference image, the walls were eliminated by removing components touching the border of the image, as the object was always located in the middle.

The resulting coarse hand segmentation served as the foreground for GrabCut, whereas the preprocessed projected point cloud of the object served as the background.

As we observed erroneous results when applying GrabCut with only a few object parts visible, the unoccluded reference object was inserted into the image by copying it to a space that was not occupied by the original object or the hand. Defining these pixels as background helped GrabCut segment parts of the object that were similar to the hand in color.

**Figure 6:**
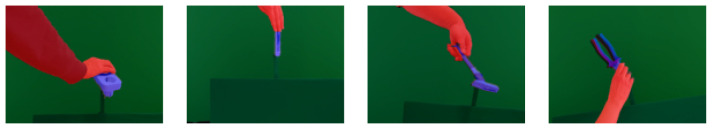
Examples of automatically generated masks for objects (blue) and hands (red) on top of raw RGB images from the dataset.

Finally, we ran the GrabCut optimization and obtained a refined segmentation of the hand. By subtracting this hand segmentation from the segmentation of the reference object, the final object segmentation mask was obtained. Figure 6 shows examples of the final instance segmentation labels overlaid on the color image.

## 3. Dataset Generation for Instance Segmentation

Before any of the generated labels could be used for training, the green screen on the input images needed to be replaced. Otherwise, segmentation could become trivial for a model specializing in the green screen. By using the captured background recordings and the foreground segmentation computed by Blender, the background of the images could easily be replaced.

### 3.1. Augmentation

For each sample of foreground, we randomly choose 20 background images of the same split. To further augment the appearance, we randomly cropped both the foreground and the background and applied random rotation and color jitter to the foreground. Afterward, the foreground and the background images were combined.

### 3.2. Combination of Foreground and Background

**Figure 7:**
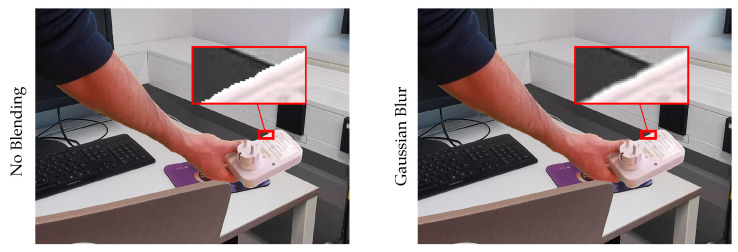
Result of using Gaussian blur on edges vs. simple overlaying of the foreground and background.

When combining the foreground and background images, special care needed to be taken at the edges where the object and hand ended and the background began. Simply stitching both color images together may result in artifacts, which could be learned by the model for easier recognition and bypassing the actual task. Therefore, specific methods, such as Gaussian blur, as employed by Dwibedi et al. [23], could be employed to blend both images. Figure 7 shows a comparison of simple overlaying and blending using Gaussian blur at the edge of the mask. Finally, the shorter edge of the combined image was resized to 448 pixels to further reduce any remaining artifacts.

### 3.3. Incorporating Thermal Data

For training on combined RGB and thermal images, the raw thermal images were registered to the RGB camera’s point of view using the depth data. To this end, reconstructed 3D points from the registered depth image were projected onto the thermal image plane using the respective intrinsic camera parameters. Thus, the thermal layer had the same size as the RGB image, but it inherited missing pixels from the incomplete depth images, which were set to zero. The registered thermal images of the foreground and background underwent the same augmentation and combination procedures as the RGB images, except for the color jittering.

### 3.4. Dataset Statistics

After generation, our dataset included 33 categories—32 object categories and the hand category. The training split contained 75,480 images with 146,912 annotations, the validation split contained 10,860 images with 21,052 annotations, and the test split contained 11,800 images with 23,067 annotations.

## 4. Experiments and Results

To demonstrate the effectiveness of our labeling and generation pipeline and establish baselines for our dataset, we trained state-of-the-art instance segmentation methods, such as Mask R-CNN [1] and PointRend [2], as well as the recently proposed, efficient YolactEdge [24]. YolactEdge achieved 61 FPS compared to 14 FPS for Mask-RCNN on an RTX 2080 Ti [24], making it a suitable choice for deployment in robotic applications. Due to their required amount of training data, we did not train Transformer-based models. PointRend is built upon Mask R-CNN and iteratively refines segmentation masks with higher resolutions, similar to rendering in computer graphics. Thus, the resulting segmentation masks were much more fine-grained than those from Mask R-CNN. In contrast, YolactEdge computed category-agnostic segmentation prototypes in parallel with bounding boxes and coefficients for combining the segmentation prototypes for each instance. Due to this parallel computation, YolactEdge was much more efficient and achieved up to 30.7 FPS with a ResNet-50 backbone on an NVIDIA Jetson AGX Xavier, which is common hardware for deployment on a mobile robot platform. We used Detectron2 [25] to train Mask R-CNN and PointRend. Unlike the default configuration, we did not freeze any part of the network. YolactEdge was trained using its officially provided code. For a fair comparison, we used a ResNet-50 [26] backbone for all three architectures. Moreover, we compared the results when transfer learning from an instance segmentation model pretrained on COCO [27] to simply using an ImageNet-pretrained [28] backbone.

Additionally, we present baseline results for incorporating thermal data. To keep it simple, we used only a four-channel input. As the pretrained weights of the first convolution only accounted for three input channels, we added a randomly initialized fourth channel for the new modality. Note that more advanced architectures and fusing methods of different modalities, such as in ESANet [11], will probably perform better.

We evaluated the performance of our trained models using the typical COCO metrics, including average precision (AP) with different intersection-over-union thresholds. The primary challenge metric, AP_50:95,_ is the mean of 10 average precision values with IoU thresholds in the range of [0.5, 0.95]. Since we are especially interested in the segmentation of the hands, we also report the AP_50:95_ for the hand category. Our results are presented in Table 2. As expected, the models pretrained on COCO outperformed those with pure ImageNet pretraining. The segmentation AP of PointRend was better, especially for higher IoU thresholds, which can be attributed to its iterative mask refinement.

**Figure 8:**
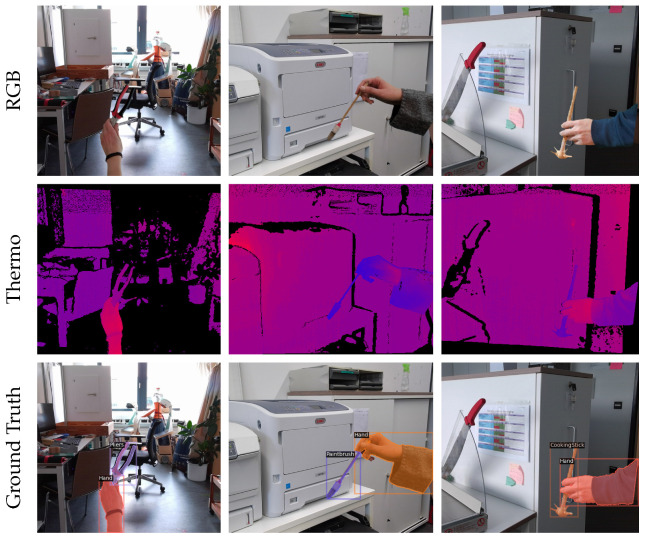
Examples of generated images for instance segmentation.

Remarkably, the performance of the much more efficient YolactEdge was on a par with that of Mask R-CNN. Training with additional thermal data further improved the segmentation AP slightly for higher IoU thresholds. Unintuitively, thermal data did not improve the ${\mathrm{AP}}_{\mathrm{Hand}}$. A possible explanation could be that due to cold hands or warm electronic devices in the background—as shown in the second column in Figure 8—distinguishing the hand from the object and background was not trivial. Therefore, we assume that using a simple four-channel input is not sufficient for effectively incorporating thermal data, and more sophisticated multi-modal architectures should be explored.

**Figure 9:**
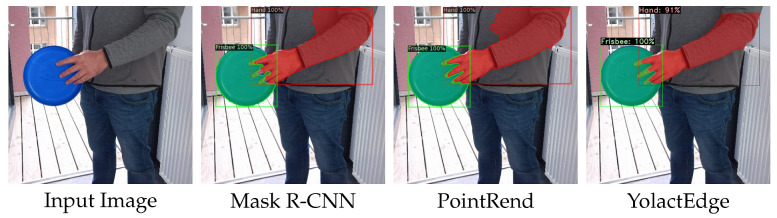
Qualitative comparison of the segmentation performance of different models (all with COCO pretraining) in a real-world setting (no stitched images).

In addition to the quantitative evaluation and to demonstrate the generalization capabilities, we also present qualitative comparisons in a real-world setting in Figure 9.

Note that our models had never seen full-sized people, and the ground-truth segmentation ended at various positions on the arm. Therefore, the segmentation performance in terms of the right side of the arm should be ignored. It can be seen that the fingers were segmented in much more detail through the iterative refinement in PointRend. As already observed in the quantitative metrics, the far more efficient YolactEdge output segmentation masks that were on a par with those of Mask R-CNN.

When grasping objects, one might only be interested in distinguishing the hand from the held object, but not the exact category of the object. Therefore, we combined all object categories and trained the instance segmentation models on only the two categories: hand and object. Such a category-agnostic instance segmentation of held objects offers the potential to operate the robot with unknown objects. This approach demonstrated improved AP compared to the multi-class problem (shown at the bottom in Table 2). In future research, we will also evaluate the performance on unseen objects.

**Figure 10:**
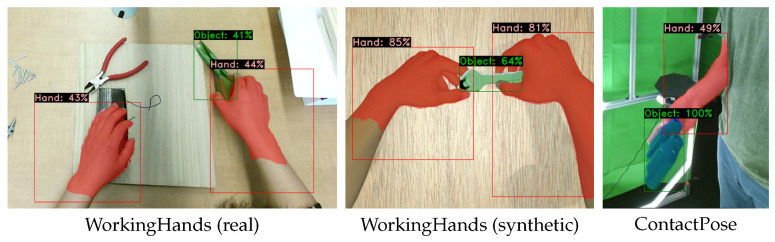
Qualitative results of cross-domain datasets generated by YolactEdge [24] trained on our OHO dataset (hand vs. object). left: applied on WorkingHands [17] (real), middle: applied on WorkingHands [17] (synthetic), right: applied on ContactPose [19].

In the last experiment, we demonstrated that the models trained on our OHO dataset did not simply focus on stitching edges by applying them to cross-domain datasets. In Figure 10, we present the qualitative results for the application of the YolactEdge model working on RGB inputs on WorkingHands [17] and ContactPose [19]. The segmentation results on both the synthetic and real-world images are impressive. The objects of known categories (wrench and scissors) were successfully detected, whereas the unknown object under the left hand in the first image was considered to belong to the background. The object in the ContactPose example was recognized, although it did not belong to the known object categories. As we only trained on samples where a hand was grasping the object, the pliers in the WorkingHands example were not detected, as they were not being grasped by a hand. If an application necessitates the detection of these objects, the reference samples in our dataset could be included in the training data to remove the bias toward grasped objects. These results show that although we recorded our dataset using a green screen, the blending of the images and the bleeding of the green screen onto the objects still resulted in trained models that could be applied to different scenarios.

## 5. Discussion

The first experiments using CNN models trained on our dataset revealed some limitations of the methods. In particular, the segmentation masks from the Mask R-CNN and YolactEdge models were of lower resolution, sometimes partially missing fingertips (see Figure 9), which was related to the network architecture. Nevertheless, there were situations in which parts of hands and objects were misclassified. This might be related to the limited diversity of the training samples in the OHO dataset. To combat this, more diverse data could be captured and automatically labeled using our label-generation pipeline. For example, by incorporating different skin colors, gloves on the hands, or more diverse backgrounds, including people, the training data could be diversified, leading to better-trained models. The data recording setup, unfortunately, requires a uniform, colored background (green screen), which is associated with many restrictions. The experiments nevertheless showed that with a good stitching method, the artifacts introduced are of minor relevance for a generalization to real-world applications. On the contrary, the background replacement improves the generalization capabilities of the networks due to the increased diversity of the samples.

On the one hand, the manual intervention during data recording is a limiting factor for scaling up the number of objects, but on the other hand, it ensures high-quality segmentation masks in 3D, as well as 2D.

## 6. Conclusions

In summary, we described how we recorded a comprehensive dataset of hand-held objects. We were able to automatically generate instance segmentation labels for our newly recorded multi-modal dataset of hand-held objects by utilizing green screen background substitution and 3D registration of previously known object models to the captured 3D point cloud data. By training the shelf segmentation networks, we achieved basic real-time capable segmentation results, which can be used in a robotic grasping pipeline. Despite achieving satisfactory segmentation results with the recorded data, for safety-critical applications involving industrial robots with the potential to harm people, the predicted segmentation mask still needs improvement. Moreover, the potential of thermal and depth data, which are already included in the dataset, needs to be further evaluated by utilizing more advanced architectures and fusing mechanisms. We hope that with these additional modalities, the robustness of segmentation methods can be improved to a level that is acceptable for real-world robotic applications.

Besides instance segmentation, the OHO dataset offers the opportunity to investigate further tasks for robotic grasping, such as object pose estimation or object shape reconstruction which, in combination with multiple modalities, make our dataset unique. The presented OHO dataset is publicly available for scientific purposes at https://www.tu-ilmenau.de/neurob/data-sets-code/oho-dataset.

## Figures and Tables

**Table 1:** Overview of existing datasets, including ours.

Dataset	#Frames	#Objects (#Categories)	Depth	Thermal
HandNet [13]	202.9 K	0	√	-
Hand-CNN [16]	40.5 K	0 *	-	-
WorkingHands [17]	7.9 K	37 (13)	√	-
EgoHands [18]	4.8 K	0	-	-
Kim et al. [14]	401 K	0	√	√
ContactPose [19]	2.5 M	25 (25) **	√	-
OHO (ours)	5.3 K	43 (32)	√	√

* contains samples from COCO, and it is not specified how many labels can be reused; ** objects are 3D-printed in blue.

**Table 2:** Evaluation of instance segmentation on the validation set of our OHO dataset. All models used a ResNet-50 backbone. The models trained on 33 categories were supposed to distinguish different objects, whereas the models trained on 2 categories were simply trained on hand vs. object. Best results by input modality are highlighted.

#Cats	Modality	Pretraining		Bounding Box			Segmentation			
				**AP_50:95_**	**AP_50_**	**AP_75_**	**AP_50:95_**	**AP_50_**	**AP_75_**	**AP_Hand_**
33	RGB	ImageNet	Mask R-CNN [1]	67.12	87.39	77.71	58.11	88.71	62.92	78.45
			YolactEdge [24]	62.93	88.42	73.69	61.69	91.15	67.19	77.78
		COCO	Mask R-CNN [1]	**73.15**	**91.60**	82.53	64.00	**93.89**	70.88	78.40
			PointRend [2]	72.46	90.18	**82.56**	**66.31**	92.96	**75.15**	**82.57**
			YolactEdge [24]	66.33	89.09	76.67	63.50	91.58	69.93	80.20
	RGB + Thermal	COCO	Mask R-CNN [1]	**73.35**	90.52	**82.84**	63.51	**92.70**	70.71	78.92
			PointRend [2]	72.75	**90.65**	82.59	**67.08**	92.18	**77.60**	**82.73**
			YolactEdge [24]	66.68	88.28	76.42	65.41	91.41	73.64	80.51
2	RGB + Thermal	COCO	Mask R-CNN [1]	**79.33**	96.57	**87.59**	70.09	97.10	79.43	78.42
			PointRend [2]	79.21	**96.69**	87.50	**74.15**	**97.53**	**83.03**	**82.34**
			YolactEdge [24]	69.31	94.21	80.08	70.59	95.94	76.52	80.13
	RGB + Thermal		Mask R-CNN [1]	**79.60**	96.64	**88.21**	70.38	96.87	79.53	78.67
			PointRend [2]	79.34	**96.92**	87.55	**75.10**	**97.53**	**85.55**	**82.87**

## Data Availability

The OHO dataset is available for scientific use at https://www.tu-ilmenau.de/neurob/data-sets-code/oho-dataset.
